# Highly efficient and sensitive patient-specific quality assurance for spot-scanned proton therapy

**DOI:** 10.1371/journal.pone.0212412

**Published:** 2019-02-14

**Authors:** J. E. Johnson, C. Beltran, H. Wan Chan Tseung, D. W. Mundy, J. J. Kruse, T. J. Whitaker, M. G. Herman, K. M. Furutani

**Affiliations:** Department of Radiation Oncology, Mayo Clinic, Rochester, Minnesota, United States of America; Emory University School of Medicine, UNITED STATES

## Abstract

The purpose of this work was to develop an end-to-end patient-specific quality assurance (QA) technique for spot-scanned proton therapy that is more sensitive and efficient than traditional approaches. The patient-specific methodology relies on independently verifying the accuracy of the delivered proton fluence and the dose calculation in the heterogeneous patient volume. A Monte Carlo dose calculation engine, which was developed in-house, recalculates a planned dose distribution on the patient CT data set to verify the dose distribution represented by the treatment planning system. The plan is then delivered in a pre-treatment setting and logs of spot position and dose monitors, which are integrated into the treatment nozzle, are recorded. A computational routine compares the delivery log to the DICOM spot map used by the Monte Carlo calculation to ensure that the delivered parameters at the machine match the calculated plan. Measurements of dose planes using independent detector arrays, which historically are the standard approach to patient-specific QA, are not performed for every patient. The nozzle-integrated detectors are rigorously validated using independent detectors in regular QA intervals. The measured data are compared to the expected delivery patterns. The dose monitor reading deviations are reported in a histogram, while the spot position discrepancies are plotted vs. spot number to facilitate independent analysis of both random and systematic deviations. Action thresholds are linked to accuracy of the commissioned delivery system. Even when plan delivery is acceptable, the Monte Carlo second check system has identified dose calculation issues which would not have been illuminated using traditional, phantom-based measurement techniques. The efficiency and sensitivity of our patient-specific QA program has been improved by implementing a procedure which independently verifies patient dose calculation accuracy and plan delivery fidelity. Such an approach to QA requires holistic integration and maintenance of patient-specific and patient-independent QA.

## Introduction

Deviations between intended and delivered radiotherapy dose distributions can potentially lead to severe clinical consequences [1]. In order to ensure treatment quality and patient safety, it is vital for comprehensive quality assurance (QA) programs to be implemented [2,3]. Patient-specific quality assurance (PSQA) procedures are an essential sub-component of such a program. The main elements of PSQA include verifying that 1) the treatment planning system (TPS) correctly models the radiation fluence produced by the treatment machine, 2) the machine performance is acceptable to deliver the intended fluence, 3) the TPS calculates an accurate representation of the dose delivered to the patient, and 4) the plan data, which is transferred between the TPS and the treatment delivery system via DICOM, is complete and interpreted correctly [4,5]. All of these potential issues are implicitly verified in traditional, end-to-end phantom measurements, which are the traditional convention for PSQA.

The phantom measurement standard evolved as a method of identifying the dosimetric deviations associated with complex intensity modulated radiation therapy (IMRT) deliveries. These modern IMRT treatments, which include small, highly modulated x-ray fields, create challenging problems for correct modeling in the TPS and delivery on the treatment machine [6,7]. The PSQA phantom measurement technique involves measuring representative portions of the dose distribution using multi-dimensional detector arrays embedded in phantoms [8,9]. These measurements are compared to the predicted doses computed in the phantom by the TPS, and the level of agreement is quantified using gamma analysis pass rates [10]. Professional organizations have established guidelines for determining if a plan is acceptable for treatment, or whether further investigation is warranted. Recently, AAPM Task Group 218 has recommended that true composite dose distributions be measured in water-like phantoms for routine IMRT QA, with an action threshold corresponding to 90% of measured points passing a gamma criteria of 3% / 2mm [11]. Regions of failures should be further investigated to assess the clinical relevance of the measured deviations.

The emergence of spot scanning proton beam technology has driven the need to develop an efficient PSQA strategy which provides assurances of patient safety and dosimetric quality in the proton domain. Despite their commonality as radiotherapy modalities, there is no requirement a priori that the PSQA programs for x-rays and protons should be identical. A PSQA program should be tailored to focus on the particular vulnerabilities of a given modality. In the case of photons, measurements in a water-like phantom provide experimental assurance that the complicated fluence patterns modeled by the TPS are correctly delivered to the patient. The accuracy of the actual *dose calculation* in the patient, however, is not explicitly evaluated on a patient-specific level by delivery into a water-like phantom. This is because water is not a good representation of heterogeneous human anatomy, and therefore the phantom dose measurement does not provide any assurance that the dose calculation is accurate near, for instance, a tumor/lung interface. Fortunately, some classes of photon dose calculation algorithms have been shown to be acceptable for use in heterogeneous media. While one-dimensional pencil beam algorithms have been shown to perform poorly near heterogeneous interfaces, multi-dimensional convolution/superposition algorithms as well as algorithms which numerically solve the linear Boltzmann transport equation have demonstrated sufficient accuracy [12,13,14]. Additionally, photon dose distributions are robust to many anatomical changes [15]. For these reasons, water-phantom-based IMRT QA without a second check of the photon dose calculation is an acceptable standard.

Conversely, the output from a spot scanning proton delivery system is the summation of many individual spots, which are easy to model. Proton plans calculated with pencil beam convolution algorithms, however, are susceptible to particle range uncertainties and to distortions in dose distributions, primarily due to the incorrect modeling of multiple scattering in inhomogeneous media. On the other hand, the Monte Carlo method handles particle transport in heterogeneous patient geometries more accurately, and is widely accepted to be the “gold standard” technique for dose calculation [16,17,18]. The limitations of analytic proton dose calculations have previously been reported by several authors. Schuemann et al. compared analytic dose calculations to Monte Carlo for 50 patients treated with a passive scattering nozzle across 5 sites, and assessed the clinical impact of the deviations [19]. Yepes et al. performed a comparative study for 525 patients treated with pencil-beam scanning across 4 treatment sites [20]. Taylor et al. compared Monte Carlo and analytic dose predictions in lung against measurements with thermoluminescent dosimeters and radiochromic film [21]. There is consensus among all these studies that discrepancies between Monte Carlo and analytic techniques can be severe in heterogeneous media. These issues cannot be detected by a PSQA program that relies heavily on dose plane measurements in a homogeneous, water-like phantom.

Due to the scarcity of beam time for quality assurance in some modern proton facilities, a process which makes most efficient use of the treatment room is desirable. Recently, new techniques have been introduced with the goal of improving the efficiency and utility of patient-specific QA. In particular, log-file analysis has been implemented in proton clinics. For the utilization of log files to be of any value, experimental verification between the log file data and the position of delivered spots at isocenter must be established [22,23]. The additional information provided by log files has been used to supplement, rather than replace, more traditional phantom-based measurements [24]. For example, log files of actual delivered spot positions have been fed back into treatment planning systems to quantify the differences between planned and delivered dose distributions [22,25,26].

In this work, we report on the methods and clinical implementation of a novel proton patient-specific QA scheme which is simultaneously more efficient and more sensitive to detecting dosimetric issues specific to proton dose calculation and delivery. Rather than relying on the limited clinical utility of a water-like phantom measurement, both the proton fluence and dose calculation in the patient are verified separately by processes specific to these purposes. The dose calculation component of the QA is checked by a GPU-accelerated Monte Carlo-based dose second check system [27,28], while the correct proton fluence is verified by spot position detectors and monitor unit chambers which are integrated into the proton delivery nozzle [29]. This constitutes a PSQA program which is more sensitive to the specific hazards of spot scanned proton therapy, yet is unique in that it does not involve measurement of dose points/planes using separate detectors placed near treatment isocenter. This reduces the overall burden on clinical resources, allowing for improved efficiency and lowering one potential barrier to increased patient access to proton therapy. We have treated over 1000 patients in the first two and a half years of clinical operation at our proton facility; the PSQA checks for the overwhelming majority of these patients have been performed with the techniques documented in this manuscript.

## Materials and methods

All patient records used in this work were reviewed retrospectively for the purpose of quality assurance/quality improvement. Our institutional review board (IRB) reviewed this study, and determined that it does not constitute research as defined under 45 CFR 46.102 and that the requirement for informed consent could be waived.

**Fig 1:**
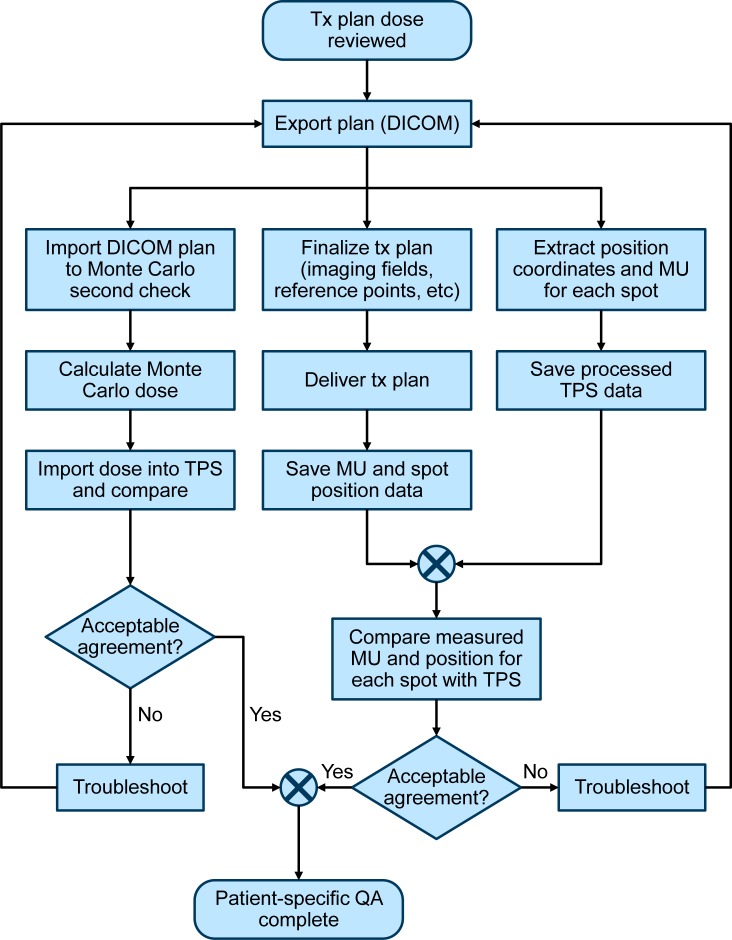
Process diagram for proton patient-specific QA. Independent checks of the dose calculation and treatment delivery system are both vital components of the process.

This patient-specific QA process consists of independently verifying the accuracy of both the calculated physical dose and the delivered proton fluence. Even though these tasks are independent, they are synergistically coupled to prevent possible data corruption or unintended changes to the treatment plan. The QA process is initiated once a planned dose distribution has been reviewed in the treatment planning system by all members of the planning team, which includes the physician, physicist, and dosimetrist (Fig 1). The plan is then exported from the planning system in DICOM ion plan format and is sent to two separate destinations: 1) the GPU-accelerated Monte Carlo-based dose second check system for immediate dose re-calculation, and 2) the log-file analysis queue for later comparison with the log files from the delivered plan. The Monte Carlo dose calculation engine, which was developed in-house, uses particle phase space files that have been pre-calculated with TOPAS [30] using detailed nozzle geometry implementations. A detailed description of this dose calculation platform and its validation can be found in a number of references [27,28,31,32]. The Monte-Carlo-calculated dose distribution is rigorously checked, using side-by-side DVH and dose profile comparisons, against the corresponding dose generated by the commercial TPS. Discrepancies large enough to raise clinical concerns, which are physician-dependent but typically on the order of 3% or greater, are discussed and subsequent action is decided. One common example of such action is re-optimization of the plan in the commercial TPS to address any localized dose discrepancies identified by the Monte Carlo calculation. Other potential interventions might include global normalization of the TPS-generated plan or adjustment of plan beam angles. In our current process, although Monte Carlo dose calculation is not directly used by the TPS optimizer, it is indirectly used to guide optimization objectives as indicated above.

The fidelity of the proton delivery system is evaluated in parallel with the aforementioned dose calculation checks. The approved plan is first transferred from the treatment planning system to the synchrotron-based proton delivery system (Hitachi PROBEAT-V). A dry run of the plan is then delivered into water jugs, which prevent the proton fluence from reaching the walls of the treatment vault. Multiple detectors, which are integrated into the nozzle, monitor the delivery dynamically. This integration allows for convenient QA delivery at the planned gantry and table angles, all without the overhead of aligning and initializing a separate measurement phantom at isocenter. There is also no additional complexity for the characterization of plans incorporating non-coplanar beams, which can sometimes present challenging geometries for true composite dose measurements in detector phantoms. The number of protons delivered per spot is regulated and verified by two separate monitor chambers working in tandem. The upstream chamber terminates the beam upon reaching the prescribed number of monitor units (MU) in the spot, while the second downstream chamber provides independent authentication of the correct dose. The lateral position (x, y) of each spot is monitored in real time by the spot position monitor (SPM), which consists of two separate multi-wire ion chambers, each containing an array of parallel wires (separated by 0.5 mm) oriented orthogonally with respect to one another. Careful beam trajectory characterization is required at commissioning to map the spot coordinates measured at the SPM to those delivered at isocenter. When the field irradiation is complete, the treatment record data is saved for subsequent analysis. The measured position and MU of each spot are compared against their intended values extracted from the DICOM plan previously exported from the TPS. The plan is considered ready for treatment if all deviations are below the machine delivery tolerances.

Spot size and beam energy, two additional parameters which complete characterization of the particle fluence exiting the delivery nozzle, are not reported in the log file QA reports. Generalized verification of these parameters is performed as a part of standard machine QA at daily, monthly, and annual intervals. Spot size is dynamically verified during delivery, but the analysis of this data has not yet been incorporated into our QA reports. Although the beam energy of each individual spot is not explicitly verified during the patient-specific QA process, the spectroscopic properties of our beamline ensure that significant energy deviations would result in spot positional deviations, triggering a beam interlock. In the worst case scenario at the highest clinical energy (~230 MeV), a 1.9% change in range would be manifest by a 1 mm spot position error [33].

Because this log-file-based technique relies critically on the accuracy of the SPM, rigorous QA is required to verify the performance of this detector. In addition to standard daily and monthly QA tests of beam output and spot positional accuracy using separate, independent detectors, a weekly QA test of the SPM detector has also been implemented. A two-dimensional plan of single-energy spots, which creates a quasi-uniform planar dose, is delivered to an ion chamber detector array (Matrixx, IBA). The range of spot positions in this plan is large enough to sample the peripheral extent of the SPM. The measured dose is compared against both the calculated Monte Carlo dose distribution and previous weekly QA measurements using gamma analysis. Log file analysis is also performed, allowing for consistency and agreement to be established between the spot position reported by the SPM and the corresponding dose delivered to isocenter.

## Results

**Fig 2:**
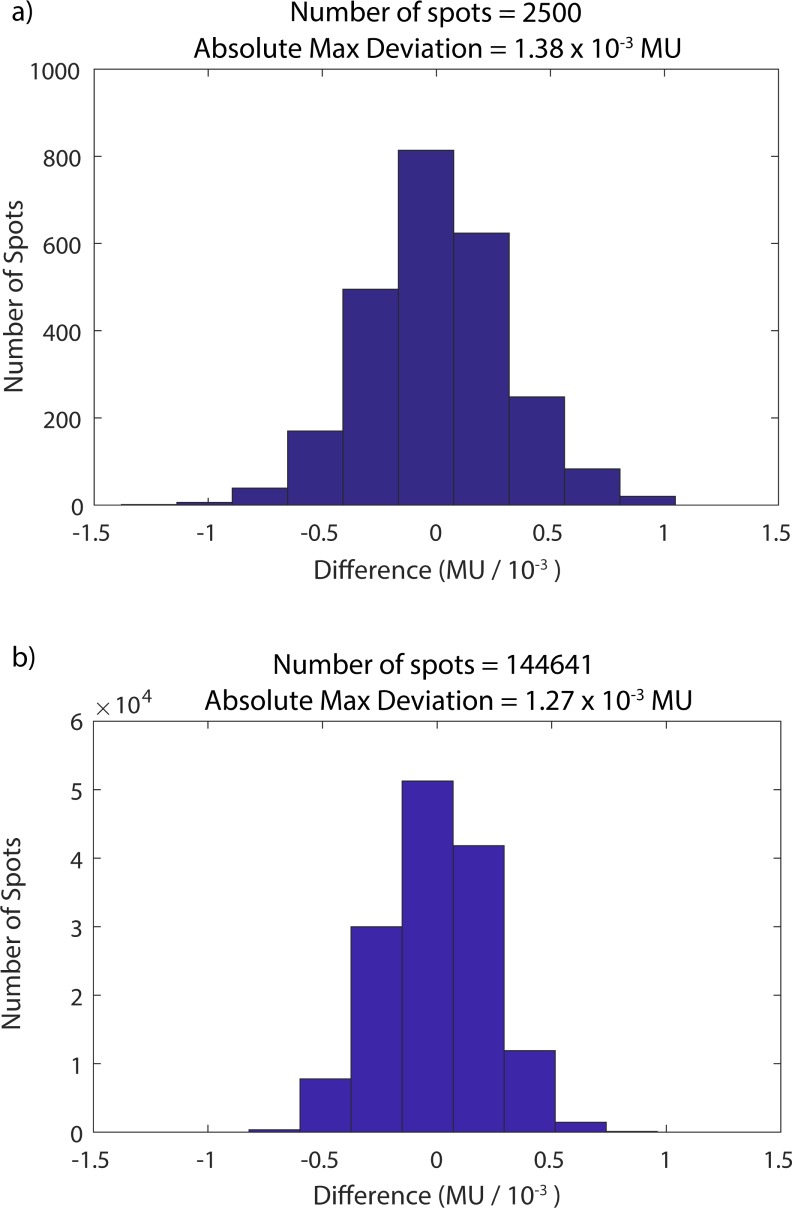
**MU difference histograms for prostate (a) and liver (b) fields.** The difference between the planned and delivered MU are analyzed spot-by-spot to verify that predefined action thresholds have not been exceeded.

Analysis is performed via a spot-by-spot comparison of the original DICOM plan with the delivered treatment record logs. Fig 2 presents histograms of the differences between the planned and delivered MU for each spot of two separate fields. Part (a) shows analysis from a prostate plan field, which contains 2500 spots and energies ranging from 159.9 to 207.5 MeV. A large liver field of 144,641 spots and energies ranging from 71.3 to 173.6 MeV is displayed in (b). Both histograms are centered about 0 MU, indicating that there is no systematic component of the error, but rather that the observed discrepancies are random in nature. The standard deviation of the MU differences are 2.2 x 10^−4^ MU and 3.1 x 10^−4^ MU for parts (a) and (b), respectively. The resolution of the dose monitor is approximately 5 x 10^−5^ MU. The absolute maximum deviations are also reported in Fig 2, indicating the closest approach of any spot in the plan to the safety abort threshold of 0.002 MU.

**Fig 3:**
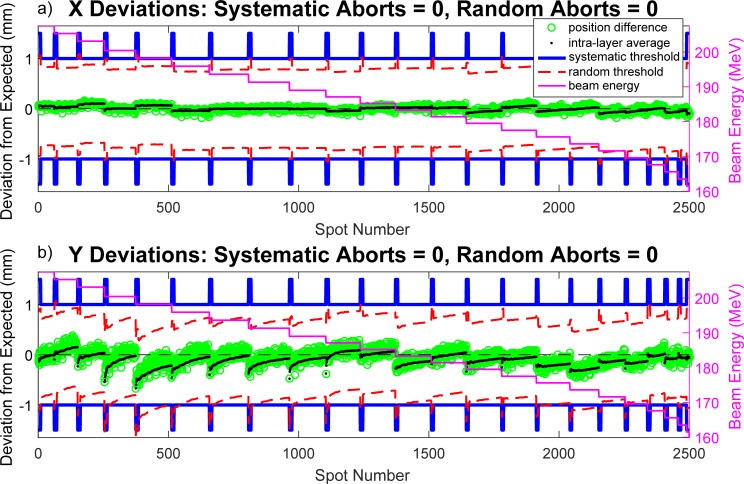
Spot position deviation analysis in the time domain. The random and systematic components of spot position deviations in both x (a) and y (b) are computed and compared to predefined action thresholds. The green circles represent the raw position deviation, while the black dots show the running average deviation in an energy spill. The tolerance for the systematic component of the error is given by the solid blue lines, which the running average deviation (black dots) should not cross. Analogously, the tolerance for the random component of the error is given by the dotted red lines (fixed expansion of the running systematic average), which the positional deviations (green circles) should not exceed.

The measured spot position data collected from the SPM is also compared with the planned values exported from the TPS. Rather than analyzing raw deviations (as is the case for the MU discrepancies shown in Fig 2), the systematic and random components of the error are computed independently. These deviations are shown in Fig 3 for each spot of a representative treatment field. The x (a) and y (b) components of each positional coordinate are analyzed and plotted independently. The systematic error term (black dots) in a given energy layer is defined as the running average difference between delivered and intended spot positions. Mathematically, this is expressed as:

$$\Delta_{sys,j}=\frac{1}{j}\sum_{i=1}^{j}{\left(x_{p}-x_{d}\right)}_{i},$$ (1)

where *x_p_* is the planned spot coordinate, *x_d_* is the delivered spot coordinate, and *Δ_sys,j_* is the running systematic deviation over the first *j* spots in the energy layer. Similarly, the random component of the error is defined as the difference between an individual spot positional deviation (delivered–intended, green circles) and the running systematic deviation:

$$\Delta_{ran,j}={\left(x_{p}-x_{d}\right)}_{j}-\Delta_{sys,j},$$ (2)

where *Δ_ran,j_* is the random component of the error of the *j*^*th*^ spot in the energy layer. The solid blue lines represent machine abort thresholds for systematic deviations, while the dotted red lines show machine abort thresholds for random deviations. The systematic tolerances are typically held constant at ±1 mm, with the exception of a small expansion (±1.5 mm) at the beginning of each energy layer. Similarly, the random tolerance is set to ±0.78 mm with an expanded tolerance of ±1.0 mm. The random and systematic tolerances were set at these levels based on internal dosimetric studies performed by the delivery system vendor to preserve acceptable dosimetric homogeneity. The expanded tolerances are primarily in place to account for the limited measurement history at the beginning of each layer, which increases the statistical uncertainty in the calculation of systematic and random deviations. The apparent positional variation of this threshold in Fig 3 is due its dependence on the variable position of the running systematic deviation (Eq 2). The beam energy associated with each layer is provided by the overlying pink trace, whose scale is referenced by the right vertical axis.

**Fig 4:**
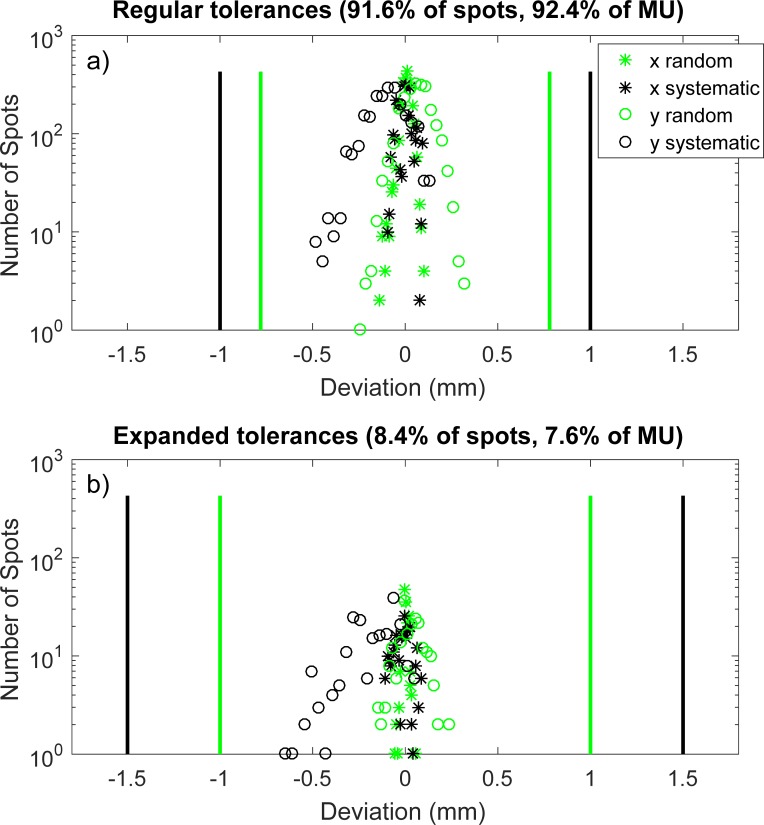
Projected spot position deviation histograms. The systematic and random components of the error (Eqs 1 and 2) are shown on log-linear histograms. Spots subject to regular tolerances (a) are separated from those governed by expanded tolerances (b).

The large volume of information presented in Fig 3 is also distilled into a more compact form as shown in Fig 4. Log-linear distributions of the random (raw deviation minus running systematic average) and systematic components of spot positional deviations in both x and y are created for all delivered spots in a given field. The points subject to the regular tolerances (a) are separated from those in the expanded tolerances (b), and these tolerances are superimposed onto each plot with black (systematic) and green (random) solid lines. Although beam energy and time domain information is lost when these errors are projected onto a single axis, easier evaluation of the magnitude of the errors with respect to abort tolerances is enabled.

**Fig 5:**
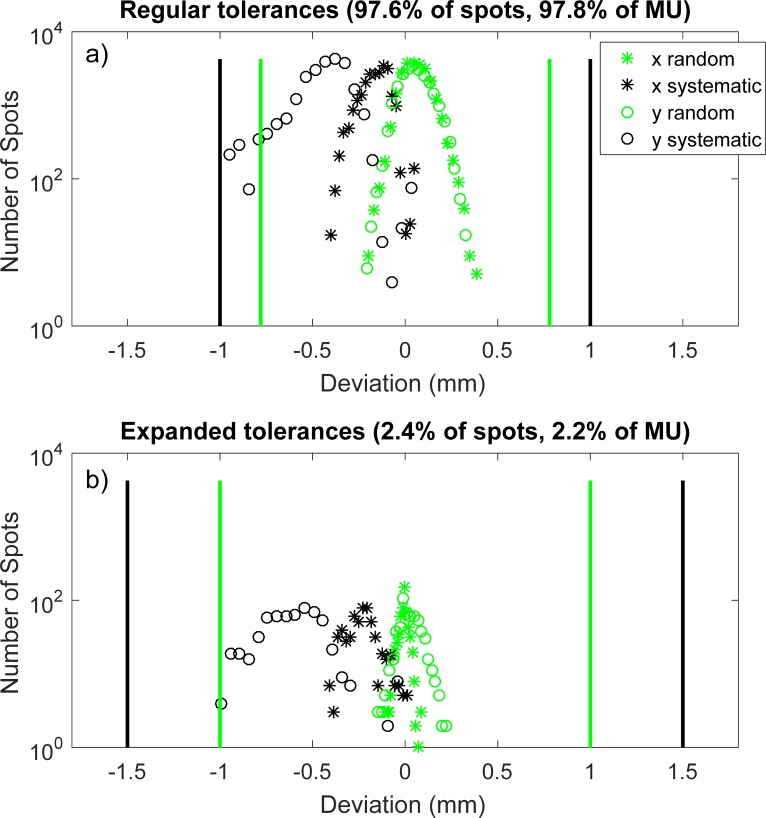
Projected spot position deviation histograms for a sub-optimal plan delivery. The systematic and random components of the error (Eqs 1 and 2) are shown on log-linear histograms. Spots subject to regular tolerances (a) are separated from those governed by expanded tolerances (b). The close approach of the y systematic data points to their corresponding tolerance (black line) suggests the potential for triggering a machine interlock during subsequent deliveries of this plan.

The spot positional deviations shown in Fig 4 lie well within their associated tolerances, indicating a high level of machine performance. In contrast, Fig 5 shows the non-ideal results of a separate patient plan which was delivered on a different day than the plan represented by Fig 4. There are a large number of systematic deviations in the y direction (black circles) which very closely approach, but do not exceed, one of the regular systematic tolerance lines in part a). This is indicative of a plan which technically passes this aspect of the patient-specific QA process, but would also trigger a deeper investigation into machine performance.

**Fig 6:**
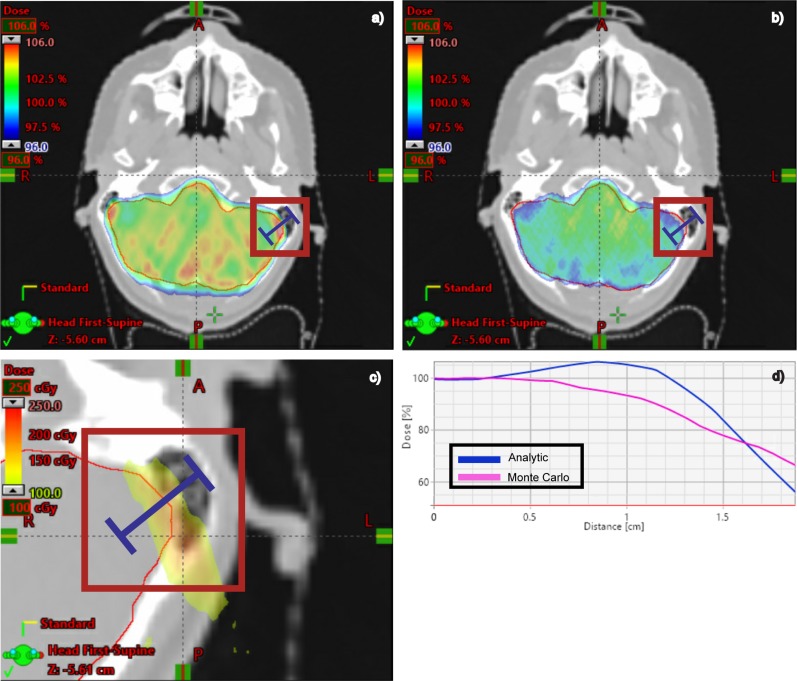
Analytic vs. Monte Carlo dose calculation comparison. Dose distributions calculated using the analytic TPS algorithm (a) are compared to those calculated with our GPU-accelerated, Monte Carlo dose second check system (b). Part (c) shows the absolute dose difference between (a) and (b) in a zoomed-in region for this 1440-cGy prescription dose. The scale bar of 100 cGy to 250 cGy corresponds to the percent range of 6.9% to 17.4%. A dose profile (d) through this region clearly shows an area of greater than 10% lower radiation dose in the target when comparing the Monte Carlo curve (pink) to the analytic TPS (blue).

The Monte Carlo dose second check system has illuminated dose calculation issues in our primary, analytic TPS which wouldn’t otherwise be detected using traditional, phantom-measurement-based QA. Fig 6 shows an example of a clinically significant, dosimetric deviation which was identified by a physicist as a part of our PSQA process. A region of the clinical target volume in the brain medial to the left mastoid air cells exhibits a greater than 10% dosimetric deficit on the Monte Carlo dose (part b) when compared to the analytic TPS calculation (part a). This difference is attributed to the difference in how scattering is modeled along the bone, tissue, and air interfaces adjacent to this region, which is treated with three beams. In addition to cases such as this, in which the observed dosimetric deviation was unexpected, there are other clinical sites such as lung in which discrepancies between analytic and Monte Carlo calculations are well-established. Regardless of this distinction, our PSQA process identifies the magnitude and location of these dosimetric discrepancies in each distinctly unique patient plan, which enables the option of performing plan re-optimization and adjustment as directed by the clinician.

Before the institution of this PSQA program, our previous process involved measuring dose planes for every treatment field at two radiological depths using the Matrixx detector array. While we have not rigorously quantified the time differences associated with our new log-file QA program, we estimate a time savings of 1) ~20 minutes due to less equipment setup/take down overhead, 2) ~10 minutes per patient plan by eliminating the need to export dose planes from the TPS and perform corresponding gamma analysis, and 3) ~2–7 minutes per field by reducing the number of times each field is delivered from two (due to dose plane measurement depths) to one. Because beam-modifying range shifters are not required to be in place for log-file QA deliveries and analysis, removing the need for the physicist to add/remove these between fields also contributes to the time savings in this final category.

## Discussion

Historically, unacceptably large failure rates have been observed in photon IMRT QA [34]. This is likely due to issues associated with modeling the delivered photon fluence as opposed to inaccuracies of the dose calculation algorithm in the water-like phantom. As radiation delivery technology and TPS modeling has improved, the frequency of plans which fail IMRT QA has decreased [35]. Nevertheless, significant challenges in modeling photon fluence persist due to difficulties in accurately modeling beam penumbra near rounded multi-leaf collimator (MLC) leaf ends and tongue-and-groove edges [36,37]. Moreover, advancements in volumetric modulated arc therapy (VMAT) and high dose rate techniques (i.e. flattening filter free modes) have resulted in additional complexity.

In contrast to the difficulty of modeling delivered photon fluence in IMRT plans, the total spot scanning proton fluence is much easier to accurately model. The proton fluence for a given field is comprised of the aggregate sum of many individual spot beamlets, each of which can be experimentally characterized and modeled in the TPS by two-dimensional profiles and depth dose curves. The profiles are a function of the beam energy and the distance along the beam central axis [38]. While proton fluence in spot-scanning systems can be well-modeled, the challenge of proton patient specific QA arises in accurately calculating the dose to the patient in the presence of heterogeneities, which affect the proton range and lateral scattering conditions [16,17,18]. Although analytic algorithms perform acceptably in many scenarios, the plan-specific nature of proton beams incident on heterogeneity interfaces means that a second check of the primary, analytic dose calculation algorithm is absolutely a necessary patient-specific quality check. The comparison of measured and computed doses in a water-like phantom provides virtually no assurance of the fidelity of an intended dose distribution in a real patient.

The QA process (Fig 1) has been designed to ensure that the intended, unaltered plan is delivered at the treatment machine. The same exported DICOM plan file that is used to generate the Monte Carlo second check dose is also used as the reference for subsequent log file analysis. This is a crucial step in the process as it benchmarks the DICOM plan reference to a specific dosimetric check of the TPS. Any subsequent, unintended plan alterations would consequently lead to dramatic failures during log file QA analysis. Examples of dangers that this process protects against include any unintended changes introduced in the TPS between the Monte Carlo dose review and final plan preparation as well as any plan transcription or interpretation errors when the plan is transferred between the TPS and the proton delivery system.

The levels which determine QA failures match the safety abort thresholds of the delivery system. The proton delivery system dynamically checks that these thresholds are not exceeded during treatment; if they are, beam delivery is interrupted. These abort threshold levels were set during the commissioning of the proton delivery system, and are based on computational planning studies [39]. Dosimetric deviations caused by MU or spot positional deviations below the appropriate abort thresholds were determined to be clinically acceptable for the beam characteristics of our facility.

In addition to assessing the deliverability of a plan, analysis of deviations in the context of abort thresholds helps provide continuing verification that the safety interlocks responsible for beam termination are functioning correctly. Moreover, it provides valuable information on machine trends, which inform downstream clinical decisions and ensure quality. Even for plans in which no QA failures are detected, routine analysis of deviations provides feedback which can be used to assess beam quality trends and for optimization of clinical margin definitions. More generally, this QA approach serves to blur the traditionally distinct lines between machine and patient-specific QA by holistically integrating machine performance tests and the analysis of patient treatment log files.

The validity of this patient-specific QA approach is dependent on the comprehensive and frequent QA of nozzle detectors with separate, independent detectors. An added benefit of this consistent SPM accuracy assurance is the continual ability to monitor machine performance during every patient treatment fraction. As such, the determination of treatment quality and safety is not only made once during the initial patient specific QA, but can be continuously checked during an entire treatment regimen. Notwithstanding our high degree of confidence in the accuracy of nozzle detectors, there is still value in periodically performing an end-to-end check of measured vs. calculated dose planes in an ion chamber array. To this end, the first two patient plans produced during each calendar week are measured (two depths for each field) with the Matrixx using these traditional techniques. This provides additional comfort in connecting our new QA paradigm with historical standards, even if these methods don’t completely address the complexities associated with accurately modeling particle range in an actual patient.

## Conclusions

In the first two and a half years of operation, our center has treated over 1000 patients, the vast majority of whose proton treatment plans have been assessed using a hybrid Monte Carlo, log-file analysis-based methodology of patient-specific QA. This process has been tailored to be more sensitive to the vulnerabilities of spot-scanned proton plans than traditional patient-specific QA methods, allowing for the identification of plan issues that would have previously remained undetected. Furthermore, the efficiency gains achieved by reducing detector setup overhead and associated analysis mechanics have allowed for resources to be channeled to other areas of clinical need, enabling higher levels of patient throughput.

## Supporting information

S1 Dataset Fig 2(A) data. Tab delimited text file containing the MU deviations for the 2500 spot field.
(TXT)

S2 Dataset Fig 2(B) data. Tab delimited text file containing the MU deviations for the 144461 spot field.
(TXT)

S3 Dataset Fig 3(A) data. Tab delimited text file containing (in order of column number) spot number, raw position deviation, running average deviation, upper systematic tolerance, lower systematic tolerance, upper random tolerance, lower random tolerance, and spot energy.
(TXT)

S4 Dataset Fig 3(B) data. Tab delimited text file containing (in order of column number) spot number, raw position deviation, running average deviation, upper systematic tolerance, lower systematic tolerance, upper random tolerance, lower random tolerance, and spot energy.
(TXT)

S5 Dataset Fig 4(A) data. Tab delimited text file containing (in order of column number) random component of positional deviation in x, random component of positional deviation in y, systematic component of positional deviation in x, and systematic component of positional deviation in y.
(TXT)

S6 Dataset Fig 4(B) data. Tab delimited text file containing (in order of column number) random component of positional deviation in x, random component of positional deviation in y, systematic component of positional deviation in x, and systematic component of positional deviation in y.
(TXT)

S7 Dataset Fig 5(A) data. Tab delimited text file containing (in order of column number) random component of positional deviation in x, random component of positional deviation in y, systematic component of positional deviation in x, and systematic component of positional deviation in y.
(TXT)

S8 Dataset Fig 5(B) data. Tab delimited text file containing (in order of column number) random component of positional deviation in x, random component of positional deviation in y, systematic component of positional deviation in x, and systematic component of positional deviation in y.
(TXT)

S9 Dataset Fig 6(A) data. Tab delimited text file containing the two-dimensional dose plane data (units of Gy, including the region of focus in part c) calculated by the analytic TPS.
(TXT)

S10 Dataset Fig 6(B) data. Tab delimited text file containing the two-dimensional dose plane data (units of Gy, including the region of focus in part c) calculated by the Monte Carlo dose calculation engine.
(TXT)
